# Assessing Environmental Risks during the Drug Development Process for Parasitic Vector-Borne Diseases: A Critical Reflection

**DOI:** 10.1021/acsinfecdis.4c00131

**Published:** 2024-03-27

**Authors:** Kayhan Ilbeigi, Carlos Barata, João Barbosa, Michael G. Bertram, Guy Caljon, Maria Paola Costi, Alexandra Kroll, Luigi Margiotta-Casaluci, Eli S.J. Thoré, Mirco Bundschuh

**Affiliations:** † Laboratory of Microbiology, Parasitology and Hygiene, University of Antwerp, 2610 Wilrijk, Belgium; ‡ Institute of Environmental Assessment and Water Research (IDAEA-CSIC), Jordi Girona 18, 08034 Barcelona, Spain; § Blue Growth Research Lab, Ghent University, Bluebridge, Wetenschapspark 1, 8400 Ostend, Belgium; ∥ Department of Wildlife, Fish, and Environmental Studies, Swedish University of Agricultural Sciences, 90187 Umeå, Sweden; ⊥ Department of Zoology, Stockholm University, Svante Arrhenius väg 18b, 114 18 Stockholm, Sweden; # School of Biological Sciences, Monash University, 25 Rainforest Walk, 3800 Melbourne, Australia; ∇ Department of Life Sciences, University of Modena and Reggio Emilia, 41125 Modena, Italy; ○ Swiss Centre for Applied Ecotoxicology, CH-8600 Dübendorf, Switzerland; ◆ Institute of Pharmaceutical Science, Faculty of Life Sciences & Medicine, King’s College London, WC2R 2LS London, United Kingdom; ☆ TRANSfarm - Science, Engineering, & Technology Group, KU Leuven, 3360 Lovenjoel, Belgium; ★ iES Landau, Institute for Environmental Sciences, RPTU Kaiserslautern-Landau, Fortstrasse 7, 76829 Landau, Germany; ▼ Department of Aquatic Sciences and Assessment, Swedish University of Agricultural Sciences, Lennart Hjelms väg 9, SWE-75007 Uppsala, Sweden

**Keywords:** drug development, environmental risk, One Health, parasitic vector-borne disease

## Abstract

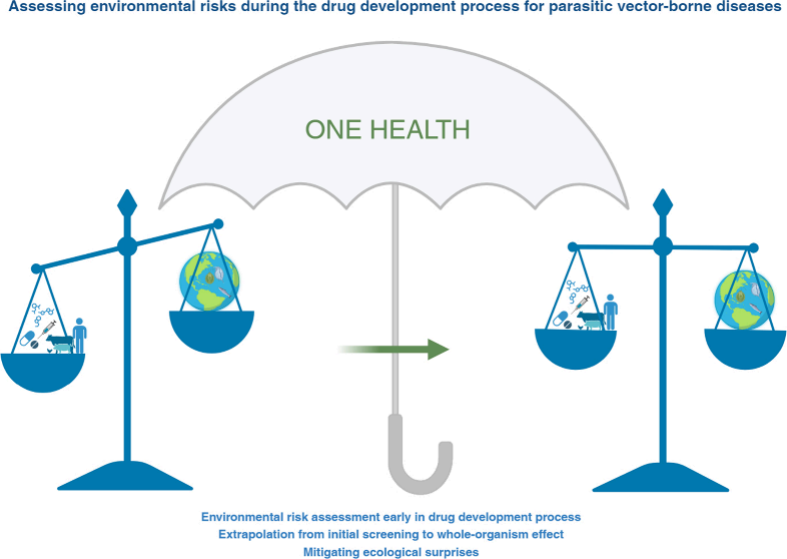

Parasitic vector-borne diseases (VBDs) represent nearly 20% of the global burden of infectious diseases. Moreover, the spread of VBDs is enhanced by global travel, urbanization, and climate change. Treatment of VBDs faces challenges due to limitations of existing drugs, as the potential for side effects in nontarget species raises significant environmental concerns. Consequently, considering environmental risks early in drug development processes is critically important. Here, we examine the environmental risk assessment process for veterinary medicinal products in the European Union and identify major gaps in the ecotoxicity data of these drugs. By highlighting the scarcity of ecotoxicological data for commonly used antiparasitic drugs, we stress the urgent need for considering the One Health concept. We advocate for employing predictive tools and nonanimal methodologies such as New Approach Methodologies at early stages of antiparasitic drug research and development. Furthermore, adopting progressive approaches to mitigate ecological risks requires the integration of nonstandard tests that account for real-world complexities and use environmentally relevant exposure scenarios. Such a strategy is vital for a sustainable drug development process as it adheres to the principles of One Health, ultimately contributing to a healthier and more sustainable world.

## Introduction

Over the last three decades, more than 30 new human pathogens have been identified, 75% of which originate from animals.^1^ These so-called vector-borne diseases (VBDs) represent 17% of the estimated global burden of infectious diseases, leading to the loss of around 700,000 human lives annually, with 80% of the world’s population being at risk of infection.^2^ Leishmaniasis, malaria, Chagas disease, human African trypanosomiasis (HAT), animal African trypanosomiasis (AAT), schistosomiasis, and babesiosis are among the most important parasitic VBDs affecting humans and animals worldwide.^2−4^ Vectors can spread pathogens from animals to humans, and vice versa. The increasing number of global travelers, growth in global trade, rapid urbanization of tropical regions, increased interactions of humans with animal pathogens and vectors in constrained environments, and climate change, in a combination with a range of other societal, cultural, and behavioral practices, have led to growing socio-economic impacts of VBDs in endemic countries and beyond.^5,6^ In addition, the limited availability of drugs, along with their high toxicity to both humans and animals, low efficacy, as well as the rapid development of drug resistance, exacerbate these challenges.^7−10^ Apart from these therapeutic issues, the environmental impacts of pharmaceutical use, mainly the active pharmaceutical ingredient (API) relative to the excipients, are of increasing concern worldwide, resulting in calls for proper consideration of environmental risks during drug development, production, use, and disposal.^11^

Active pharmaceutical ingredients (APIs), along with their metabolites and other transformation products, enter the environment throughout a drug’s lifecycle, for example, through industrial and hospital effluent, domestic wastewater treatment plant (WWTP) effluent, and animal waste runoff.^12^ Consequently, residues of APIs and their breakdown products are a prominent group contributing to the growing global chemical pollution crisis.^10,13^ These chemicals are designed and selected to elicit biological reactions by interacting with molecular targets that can be evolutionarily conserved across various taxa.^14,15^ The higher the degree of interspecies conservation, the higher the risk of eliciting unintended pharmacological effects in nontarget organisms exposed to these compounds.^16^ Because drugs are designed or selected to be highly potent and specific for targets or pathways, in some cases they can elicit unwanted effects in wildlife even at environmentally relevant concentrations (e.g., in the ng/L to μg/L range), which makes the presence of these drugs in the environment concerning.^17^ While the contamination of the aquatic ecosystem occurs through drug excretion and improper disposal,^18^ the terrestrial environment is also exposed to APIs through the application of sewage sludge, leaching from landfills, the application of treated or untreated wastewater to irrigate arable land, and directly from excretion of veterinary medicines by animals.^19^ As a noteworthy illustration of aquatic pollution, the upregulation of vitellogenin, a protein predominantly associated with females, in male fish exposed to estrogenic APIs in the environment resulted in the feminization of freshwater species.^20,21^ A striking case in the terrestrial environment is that of unforeseen secondary poisoning effects following off-label use of the nonsteroidal anti-inflammatory drug diclofenac, which had a devastating impact on vultures and caused a >99% population decline among *Gyps* vulture species in India and Pakistan due to renal failure after the consumption of diclofenac-contaminated cattle carcasses.^22,23^ Additionally, the ramifications of improper disposal are evident in cases like ivermectin, which has been detected in soil and water, potentially serving as a source of single- or multidrug resistance.^24^

To minimize ecological risk, the European Union (EU) and the United States (US) have developed regulatory protocols that require new drugs to undergo an environmental risk assessment (ERA), which typically coincides with Phase III clinical trials before being granted authorization to enter the market. Additionally, it is noteworthy that the requirement for chronic ecotoxicity testing for human medicines was only introduced in the EU in 2006 and has not been universally mandated in the US.^25,26^ As a result, most of the legacy drugs registered before 2006 are lacking chronic ecotoxicity data, leading to a mere 12% of all drugs having a comprehensive set of ecotoxicity data.^15^ For example, on the German market, ERA data are absent for 281 out of 404 APIs used in human medicines pointing to unknown environmental risks.^27^ Risks may be identified if the expected environmental concentrations exceed 0.01 μg/L (as inferred from consumption data) or due to specific substance characteristics, such as endocrine activity.^27^ Drug pollution risks and their impacts on animals and the environment have been largely ignored before 2006,^28^ strongly supporting the relevance of the One Health principle. Consequently, considering One Health early in the drug development process is essential for a truly sustainable society.

The necessary improvements in the regulatory ERA of human pharmaceuticals have been recently summarized.^27^ The new EU pharmaceuticals strategy for Europe^29^ takes some of these considerations into account, as reflected in the new Directive and Regulation, which revises and replaces the existing general pharmaceutical legislation, adopted by the European Commission on 26 April 2023.^29^ In contrast, the Regulation defining the authorization of veterinary medicines, (EU) 2019/6 (veterinary medicine product regulation, VMPR), has not been updated. According to this legislation, ERAs “should be” mandatory for all new veterinary medicine products placed on the market (Recital 31), specific requirements for GMOs apply (Article 8), and existing products should be assessed in case of the API being potentially harmful (Article 72), with details listed in Annex II of (EU) 2019/6. The ongoing EU Partnership for the Assessment of Risks from Chemicals (PARC)^30^ is dedicated to suggesting improvements to the ERA for chemicals currently regulated under the veterinary medicine product regulation, among others.

In the context of parasitic VBDs, several antiparasitic drugs have been developed. Typical examples of major VBD drug classes include nitroimidazoles (for protozoa), benzimidazoles (for nematodes), praziquantel (for trematodes), and aminoquinolines (for Apicomplexa, such as *Plasmodium*). While ecotoxicological data are available for some of these drugs, such as metronidazole,^31−34^ albendazole,^35,36^ and praziquantel,^37^ such data are either limited or lacking for others, highlighting the absence of a One Health perspective even for widely used antiparasitic drugs. For instance, benzimidazoles bind to parasite β-tubulin, a protein that is highly conserved among eukaryotes, and interfere with microtubule polymerization^38−40^ pointing to the susceptibility of nontarget organisms. In Germany, for example, antiparasitic drugs together with antibiotics account for about 90% of the veterinary medicinal products (VMPs) used in livestock production.^41^ It is clear that antiparasitic drugs should be assessed for potential risks for nontarget organisms before they are released into the environment, minimizing the possibility of adverse ecological effects.

In this contribution, we communicate insights into an often overlooked aspect of the One Health tripartite approach: the environment. We specifically focus on the potential environmental risks posed by antiparasitic drugs. Further, we provide an overview of the ERA procedure for VMPs, and we emphasize the significance of incorporating environmental risk considerations early in the drug development process. Finally, we discuss the extrapolation from initial screening to whole-organism effects and outline strategies to better predict and mitigate potential ecological risks of pharmaceutical use.

## Environmental Risk Assessment for Veterinary Medicinal Products

**Figure 1:**
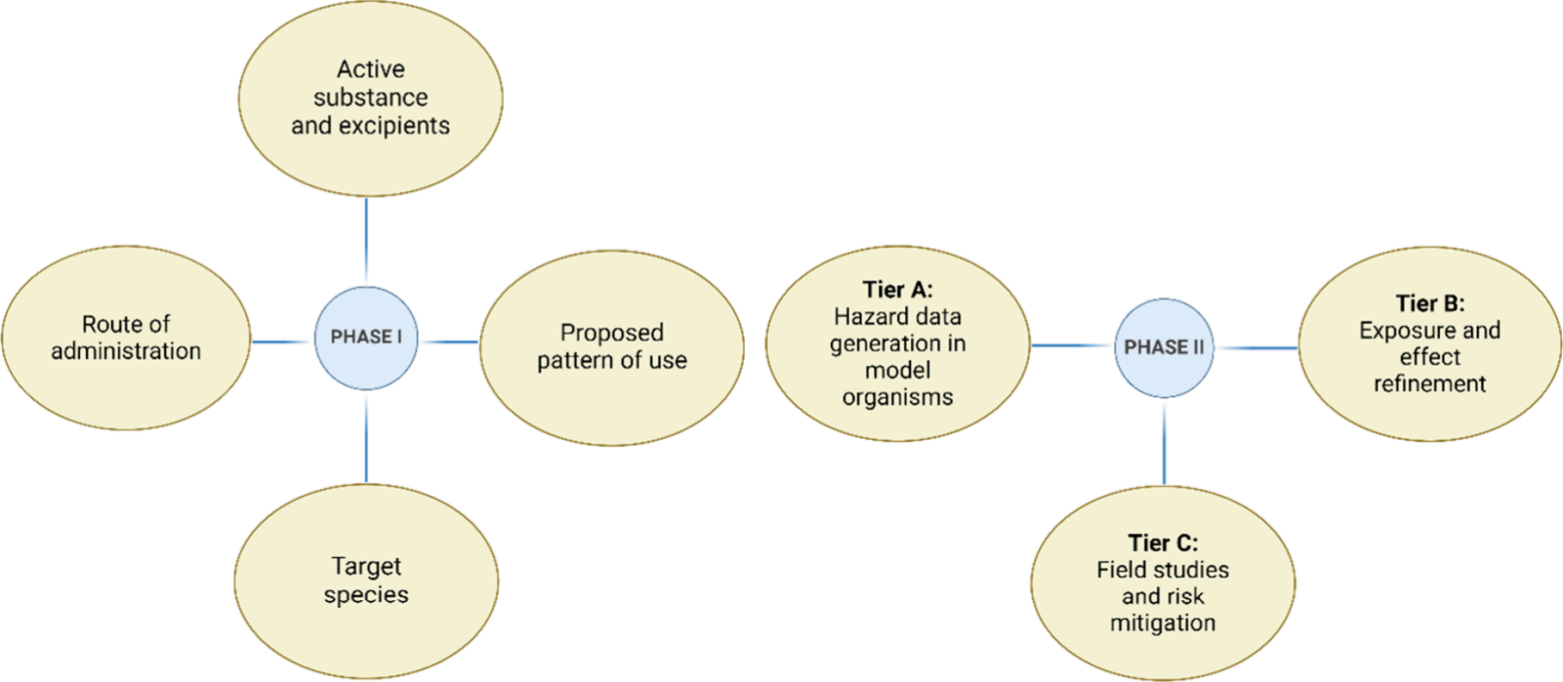
Summarized schematic of Phase I and Phase II of environmental risk assessments for veterinary medicinal products.

In the environmental risk assessment (ERA) of VMPs, the European Medicines Agency’s (EMA) guidelines advocate a tiered approach outlined in VICH guidelines 6 and 38 (Figure 1).^42,43^ The process begins with Phase I, which involves a thorough evaluation of the product’s environmental exposure, focusing on its physiochemical characteristics, usage, dosing, and excretion pathways. The decision to progress to Phase II, as outlined in VICH guideline 38, is contingent upon the findings from Phase I, particularly questions 1–19.^44^ Veterinary medicinal products with limited application and minimal environmental exposure are typically contained within Phase I, excluding the need for further analysis. Notably, Phase I assessments exclude product lifecycle stages such as manufacturing and disposal and only consider exposure resulting from product use. Products that conclude in Phase I typically include those used for individual animals or companion animals, or those with predicted environmental concentrations (PECs) below established thresholds (e.g., PEC_soil_ ≥ 100 μg/kg).^45^

Veterinary medicinal products flagged in Phase I for their potential environmental impact undergo a more extensive evaluation in Phase II.^46^ This involves comparing the product’s PEC against the lowest effective concentration derived from standard ecotoxicity tests in environments like soil and water. Phase II, further divided into tiers A–C, intensifies the evaluation. Tier A generates hazard data in model organisms to calculate the predicted no-effect concentration (PNEC), with PECs calculated under worst-case scenarios for various environmental compartments. A PEC/PNEC ratio exceeding 1 triggers more in-depth studies in Tier B, refining both PEC and PNEC values through fate and effect studies. In Tier C, if risks are identified for a specific compartment, field studies or risk mitigation measures are considered. This phase accounts for environmental processes such as hydrolysis, photolysis, and biodegradation (mineralization and biotransformation) of a VMP, refining initial findings from short-term laboratory ecotoxicity data with longer-term, semifield, or field data.^41,46^

The environmental risk assessment’s outcome is crucial for the VMP’s approval process. If risks are identified and cannot be mitigated, the environmental risks are weighed against the VMP’s benefits. Approval is granted if the benefits outweigh the risks. This comprehensive evaluation extends to VMPs containing multiple active substances, aiming at safeguarding the environment.^47^ A recent reflection paper on ERA for VMPs used in companion animals highlights two key concerns: historically, the environmental impact of these products has been considered negligible, often bypassing a Phase II ERA.^48^ Additionally, the Phase I assessment lacks requirements for data determining environmental fate.^48^ This is particularly concerning given the rising number of companion animals in Europe, potentially increasing environmental exposure to these products.^48^ Moreover, the necessity to develop new drugs for VBDs coupled with the emergence of these diseases in new regions underscores the importance of considering potential risks at an early stage in the process. This is particularly crucial in scenarios where ERA might be overlooked due to use-based exemptions.

## Considering Environmental Risks during Drug Development

Because risk assessments are required for regulatory submissions (i.e., marketing approvals and line extensions), environmental data generation for new veterinary drugs typically starts late during the drug development process, that is, during Phase I to III of clinical trials, and thus only after a significant investment has already been made in terms of time and resources. Nonetheless, the environmental perspective can be considered earlier in the drug development process. Environmental risks may be judged based on characteristics of a drug candidate such as mode of action, potency, specificity, dose, chemical structure, stability, and ADME (absorption, distribution, metabolism, and excretion). The four main drivers of environmental risk are environmental persistence (resistance to environmental degradation), mobility (due to hydrophilicity and structural properties), bioaccumulation (due to lipophilicity and ADME profile in wild species), and ecotoxicity (negative effects on nontarget species).

Consequently, improving predictive tools for environmental risks to evaluate compounds *in silico* (i.e., before synthesis) is an important approach. Here, we refer to three tools (Table 1).

**Table 1:** Predictive Tools for the *In Silico* Evaluation of Drugs

tool	description	reference
(i) Estimation Program Interface (EPI) Suite Data program (EPI Suite)	provides quantitative structure–property relationship (QSPR) models to predict a variety of key chemical properties, fate and transport parameters, and acute and chronic toxicity thresholds to aquatic organisms based on chemical structure	(49)
(ii) Toxicity Forecaster (ToxCast)	compiles high-throughput *in vitro* toxicity data targeting lower levels of biological organization that are often subsequently linked to apical end points via adverse outcome pathways (AOPs); newly developed chemicals can be screened in ToxCast based on structural data	(50)
(iii) Sequence Alignment to Predict Across Species Susceptibility (SeqAPASS)	uses protein sequence alignment information to evaluate sequence similarity among a wide array of species and to estimate susceptibility of nontarget species	(51)

The characteristics for which new active compounds are optimized (efficacy, safety, balanced ADME, etc.) are interrelated with each other and also directly inform their environmental properties. For example, this means that design efforts aimed at minimizing nontarget effects and general toxicity in patients should also reduce the likelihood of similar effects on homologues in wild species. However, there are a few potential challenges: (i) target-related ecotoxicity may be unavoidable for many modes of action; (ii) some nontarget effects may not be known early in the drug development process and related ecotoxicological effects can thus not be pre-empted; and (iii) while sequence information on the preservation of drug targets in wild species exist, this is not comprehensively incorporated in *in silico* prediction of off-target effects on orthologues. Solutions might include developing activity assays using wild species orthologues and using new technologies such as reporter gene assays to accelerate assay development, utilizing existing research and development data for early prediction of ecotoxicological potential, and adapting existing tools (e.g., *in vitro* cytotoxicity assays, off-target screening panels, genotoxicity, and short-term rodent toxicity) for environmental read-outs. Another plausible route toward reduced environmental pollution and therefore reduced environmental risk potential of any compound is better degradability. Compounds that are susceptible to mechanisms of (biotic or abiotic) environmental degradation demonstrate shorter environmental half-lives (reduced persistence) and therefore overall lower environmental concentrations, unless more toxic transformation products are formed.^52,53^

## Extrapolation from Initial Screening to Whole-Organism Effects

As mentioned earlier, it is crucial to apply reliable approaches (e.g., development of receptor-based assays) during the drug development process. Accordingly, studying the mechanism of action of chemicals across biological hierarchical levels can be synergistically harmonized with the adverse outcome pathway framework.^54^ Describing toxicity mechanisms from molecular initiating events to apical adverse outcomes, the application of the adverse outcome pathway concept facilitates toxicity assessment of APIs.^55^ Nonetheless, adverse outcome pathways themselves encounter certain drawbacks, which need to be improved: (i) existing AOPs predominantly emphasize implications for human health only and (ii) do not consider real-world circumstances; (iii) AOPs are not complementary to the already existing mode-of-action data; (iv) there is a demand for deeper biological understanding of effects on organisms within ecosystems; and (v) AOPs often do not include effects beyond individuals, while the protection of populations is the target of ERAs. The AOP framework was designed to bridge the gap between measurable pathway perturbations in high-throughput screening assays and broader impacts on survival, growth, and reproduction. However, while the AOP concept aims to address this disparity and still has the potential to contribute to the drug discovery process,^56^ it often lacks well-developed and quantitatively relevant AOPs for risk assessment, hindering its effectiveness.^54^

New Approach Methodologies (NAMs) offer a potential solution to improve AOPs. By definition, an NAM is “a broadly descriptive reference to any technology, methodology, approach, or combination thereof that can be used to provide information on chemical hazard and risk assessment that avoids the use of intact animals (all vertebrates and few invertebrates such as Cephalopoda)”.^57^ New Approach Methodologies are seen as a promising solution to address the limitations of the existing approaches during the drug development process, which often rely on laboratory experiments and nonanimal studies (*in vitro* tests with cells, receptors, enzymes, etc., vertebrate embryos, most invertebrates) to gather data that supports the safety of a drug before entering the clinical trial phase and eventual market release (i.e., authorization). These advanced methodologies include a wide range of techniques employed in toxicogenomics, bioinformatics, system biology, and computational toxicology. Using NAMs for ecotoxicology includes enhancing physiologically based pharmacokinetic (PBPK) models, increasing access to multiomics, and scalability of fish *in vitro* systems, which can help bridge the disciplinary separation between human and environmental health. Moreover, it can provide the opportunity to improve AOPs by including cost-effective ecologically relevant assays that consider the key features of the organisms. For example, performing short ecotoxicological assays on *Daphnia* using existing OECD tests and design experiments, which are equally informative and more ecologically relevant than the existing design.^58^ Additionally, following the previous mentioned assays it is possible to screen chemicals according to their ecotoxicological mode-of-action^59^ (i.e., toxic anorexia, endocrine disruption, metabolic assays and teratogenesis) and use OMIC methods to study the mechanism of action of drugs (transcriptomic, metabolomic-lipidomic, functional biomarker assays, enzymatic assays, immunochemistry, histopathology, physiological assays, etc.), which are other approaches that can potentially improve AOPs. Some examples of applying NAMs include using zebrafish larvae as a model to assess the impact of drugs on the cardiovascular^60^ and nervous systems.^61^ Therefore, incorporating NAMs into the regulatory framework paves the way for more efficient and comprehensive evaluations of the ecological risks of drugs and promises to facilitate our efforts to more effectively protect the environment and its inhabitants.

## Mitigating Ecological Surprises: A Forward-Looking Approach

While the strategies described above are indeed helpful in prioritizing efforts and aiding ecotoxicological risk assessments, it should be clearly noted that these approaches can only be seen as one piece of the puzzle when assessing environmental risks or hazards. Being limited to suborganismal responses or acute and chronic responses of a few standard test species ignores the wide diversity of other species, and even more importantly, the interactions between them, as well as the ecosystem functions these organisms provide. Consequently, even if not required by current regulations, nonstandard tests that move toward the assessment of responses at higher levels of biological complexity, incorporate an environmentally relevant exposure profile of the drug in focus, and can play a central role.

Standard ecotoxicological tests that conform to internationally accepted guidelines, such as those outlined by the OECD, may fall short in providing a comprehensive understanding of the prolonged, low-dose exposure scenarios that are typical of drug pollutants. Those exposure scenarios could expand over multiple generations, with partly unpredictable changes in responeses of test organisms among generations.^62^ These tests primarily focus on the toxic effects of chemicals, such as mortality, reproductive perturbations, and developmental impacts, such that we risk overlooking subtler (sometimes difficult to repeatably quantify) yet ecologically relevant effects, including changes in animal behavior.^63−66^ Exposure to a wide range of chemicals, including drugs, has been shown to induce behavioral changes in diverse species, which can have far-reaching consequences for individual fitness and species interaction with consequences on ecosystem integrity.^67^

Further, while traditional ecotoxicological assessments serve as a valuable tool for establishing safe concentration limits of individual chemical substances, it is important to recognize that organisms in their natural habitats typically face exposure to a complex mixture of chemicals, including multiple drugs. Despite the fact that certain drugs are known to have dangerous interactions with humans and are therefore not prescribed together, such mixtures nevertheless find their way into the environment. Failure to account for these interactive effects in ecotoxicological assessments can lead to inaccurate evaluations of the ecological impact of pharmaceutical pollutants and the formulation of concentration levels, such as environmental quality standards (EQSs), that are not sufficiently protective for the environment.^68^

Adding to this complexity is the controlled environment in which test organisms are typically studied, which may not be representative for the mixed-stressor environment they encounter in the wild.^69^ For example, wildlife typically experiences high levels of resource competition, predation pressure, and parasitic infections, along with various other sources of stress. While it is not feasible to test every possible combination of drugs under various environmental conditions, environmental chemists and ecotoxicologists are shifting their focus from solely assessing the toxicity of individual chemicals. Instead, they are now also characterizing the effects of complex chemical mixtures within diverse indoor and outdoor settings.^70^ This shift aims to enhance the relevance and precision of ecological risk assessments of chemical mixtures including mixtures of drugs.

These nonstandard testing strategies, in combination with the standardized approaches, support the assessment of environmental concerns more retrospectively and thus after authorization of drugs. In Europe, the Water Framework Directive (WFD)^71^ and the Priority Substance Directive^72^ establish a structure to recognize substances that could potentially be risky for freshwater ecosystems. These directives also lay a legal foundation wherein member countries are obliged to oversee and adhere to the EQSs set for these substances. When concentrations of these substances in surface waters exceed EQSs, a range of measures can be adopted to reduce the concentrations to acceptable levels. To determine the most effective course of action while preserving the societal advantages of the substance as much as possible, a comprehensive understanding of the substance’s emission, exposure, fate, and effect is crucial, which can be provided via a comprehensive ERA. This includes pinpointing all significant sources and evaluating the size of their contributions.^73^ Therefore, ensuring that the existing ERA covers the Directive aims for “good status” of all ground and surface waters and identifying the lowest EQS are crucial to have a more synchronized approach. The importance of such evaluations is clearer when we look into the real-life examples of substances such as diclofenac, ibuprofen, and erythromycin.^74^ A recent review has put forth some recommendations aimed at improving environmental risk management strategies for human pharmaceuticals, such as integrating ERA conditions with a focus on thoroughness and effective risk mitigation measures, developing an interconnected and coordinated approach within legislative frameworks to ensure environmental protection, and maintaining complete, current, and transparent environmental data.^27^ These proposed improvements, while initially targeted at human pharmaceuticals, can be potentially applicable in the context of VMPs.

## Reflection

The complex interaction between drug development for parasitic VBDs and their environmental consequences presents various challenges, from drug biodegradation potential and accumulation in organisms to potential toxicity and biological effects in ecosystems. This emphasizes the need for robust methodologies and comprehensive approaches to ensure that drug development not only benefits human and animal health but also safeguards environmental integrity.

We strongly support the urgency of integrating environmental considerations early in the drug development process for parasitic VBDs to ensure not only human health but also the health of animals and environmental integrity. This approach is not to limit the development of promising drugs but to proactively identify and mitigate potential environmental risks. Such integration involves embedding environmental expertise in research and development projects, performing *in silico* assessments before compound synthesis, developing and applying predictive tools like the EPI Suite, ToxCast, and SeqAPASS, and using NAMs. The need to deprioritize compounds with high potential environmental impact could be a proactive approach implemented by regulators and companies. That being said, it seems crucial to balance these environmental considerations with the need to ensure access to medicines, especially in developing countries where such access is rather limited. While the shift of some European countries toward integrating environmental safety data into the marketing authorization process is promising, it is essential that this does not limit the availability of life-saving drugs. This means that the potential environmental risk must be significant and evidence-based to allow a realistic decision. The use of NAMs and modeling for early hazard assessments, although predictive, can provide valuable time to implement anticipatory risk mitigation strategies without prematurely stopping the development of promising drugs.

Going forward, a transformative approach that includes an environmental impact assessment of the risk–benefit considerations made during drug development is necessary. While ERA is mandatory for submission in many global regions, environmental testing is currently started during the later stages of the development process, after compound design is concluded and a final drug candidate has been selected. In order for environmental properties to be taken into consideration in the design of new actives and the selection of drug candidates, environmental data generation and environmental risk evaluations need to be considered earlier in the drug research and development process. Therefore, an interdisciplinary approach that integrates One Health perspectives plays a pivotal role. This includes collaboration among environmental scientists, toxicologists, and medicinal chemists, alongside continued research and innovation in refining NAMs, increasing their predictive accuracy, reliability, and reproducibility, and expanding their applicability to a broader range of environmental impacts. Although cross-species extrapolation is inherent in the ERA, more reliable ways need to be found to avoid or at least minimize ecological surprises, such as the high vulture mortality exposed to diclofenac in India. Moreover, it is crucial to extend drug development studies by environmental and toxicological training to raise the awareness of practitioners in the field. These forward-looking steps can pave the way for a sustainable drug development process, which aligns with EU actions to address the environmental challenges of pharmaceutical (veterinary) products such as the Pharmaceutical Strategy for Europe^75^ and the EU strategic approach to drugs in the environment^76^ as well as activities such as the review of the Urban Waste Water Treatment Directive and evaluation of the Sewage Sludge Directive.

In conclusion, the present communication highlights potential approaches for R&D to ensure an integrated approach for human and animal well-being, and environmental health under the One Health umbrella. The current ERA during drug discovery provides valuable insights but occurs relatively late in the process. We believe it is essential to consider environmental parameters earlier, which can be achieved by using predictive tools or NAMs. However, it is also crucial to consider that these methods primarily focus on single-substances, suborganismal responses and standard test species, and nonstandard testing strategies that incorporate more real-world complexities and environmentally relevant exposure scenarios. More importantly, while it is clear that early consideration of environmental toxicity is both doable and desirable, it should not prevent the development and availability of life-saving drugs. Instead, the focus should be on spending more time for the development of comprehensive risk mitigation strategies. By implementing these proactive measures, the pharmaceutical industry can harmonize drug development with environmental preservation, which aligns with overarching directives like the WFD and supports a path toward a healthier world.
